# A Pre and Post Survey to Determine Effectiveness of a Dietitian-Based Nutrition Education Strategy on Fruit and Vegetable Intake and Energy Intake among Adults

**DOI:** 10.3390/nu8030127

**Published:** 2016-02-29

**Authors:** Dhandevi Pem, Suress Bhagwant, Rajesh Jeewon

**Affiliations:** 1 Department of Health Sciences, Faculty of Science, University of Mauritius, Réduit 80837, Mauritius; dhandevi.pem@gmail.com; 2 Department of Marine & Ocean Science, Fisheries & Mariculture, Faculty of Ocean Studies, University of Mauritius, Réduit 80837, Mauritius; shyamb@uom.ac.mu

**Keywords:** fruit and vegetable, energy intake, nutrition knowledge, body mass index

## Abstract

The purpose of the study was to evaluate the effectiveness of a multicomponent nutrition education program among adults. A pretest—posttest design was used assessing Nutritional Knowledge (NK), BMI, Energy Intake (EI), Physical Activity Level (PAL), Dietary Intake (DI) and attitudes. 353 adults aged 19–55 years (178 control group (CG) and 175 intervention group (IG)) were recruited. IG participants attended nutrition education sessions evaluated through a post-test given at the end of the 12-week program. Statistical tests performed revealed that compared to CG, participants in IG increased fruit intake and decreased intake of snacks high in sugar and fat significantly (*p* < 0.05). NK and attitudinal scores also increased significantly in the IG (*p* < 0.05). No intervention effect was found for vegetables intake, EI, BMI and PAL (*p* > 0.05). Factors influencing NK were age, gender and education level. “Taste” was the main barrier to the application of the nutrition education strategy. Findings are helpful to health practitioners in designing their intervention programs.

## 1. Introduction

The current epidemics of non-communicable diseases (NCDs) in Mauritius are leading public health issues [1]. Recent estimates from successive National NCD Surveys at five-six years intervals show a rising trend in the prevalence of diabetes mellitus and obesity over the period 1998–2009 [2], reaching around 20.5% and 19.1%, respectively, in 2015 [3]. It has been found that the diet of Mauritians itself is one of the main problems with its high salt content and low fruit and vegetable content [1,4]. Sufficient intake of fruit and vegetable has been associated with a reduced risk of chronic diseases [5]. Studies also proposed that energy density of the diet can be lowered by adding fruit and vegetable and hence maintain body weight over time [6]. The World Health Organization and Food and Agriculture of the United Nation report recommend that adults consume at least five servings or a minimum of 400 g of fruit and vegetable per day excluding starchy vegetables [7]. Existing data suggests that despite their protective effects, fruit and vegetable intake are still inadequate in many countries [8,9] as well as in Mauritius [10]. Between the year 2002 and 2003, 89.4% and 89.6% males and females respectively were reported to have a low consumption of fruits and vegetables (FV) in Mauritius [11] and in 2009, although the availability of FV was 112.9 kg/capita/year, 198,000 obesity cases were registered [12]. In 2013, average daily fruit consumption (excluding wine) and daily vegetable consumption in Mauritius was estimated to be 30.7 g and 75.3 g respectively [13]. In 2014, availability of fruit and vegetable was approximately 362 g [14]. Messages about food are accessible by a variety of media sources and are common on the Internet, but are often confusing [15]. Hence, nutrition education research which is a dynamic field focused on communicating science-based findings is of utmost importance [16]. Reports in the literature have documented an array of models, sets of assumptions and principles, theories, and explanations that make up the adult learning knowledge base however there is no single theory of learning that can be applied to all adults. Adult learning theories describe ways in which adults assimilate knowledge, skills and attitudes [17]. Nutrition education, including its theoretical basis, should effectively address all of these issues to produce healthy individuals. Diverse settings such as workplaces, supermarkets, worksite canteens and community based studies have been used in order to understand what works best for specific groups of people [18,19]. Even though findings from such interventions are intricate to extrapolate to other settings, majority of the interventions were successful in increasing fruit and vegetable intake and improving health indicators among adults [20]. Research on interventions in adults found that those which included interpersonal component such as face to face education had reliable and constant positive effects [21]. In California for every $1 invested in nutrition education, between $3.67 and $8.34 is saved in health care costs for low income families [22]. Overall nutrition education contributes significantly to a change in nutrition related behaviors and is considered as a good investment in terms of cost benefit ratio. Rapid improvement in trends of nutrition education can be seen in other countries [23], however, though many nutrition education interventions promoted fruit and vegetable intake, relatively few efforts have targeted adults and lacked investigation of attitudes, barriers and motivators of the intervention [24,25]. To date, there are no related studies on fruit and vegetable intake and the impact of a nutrition education intervention among adults in Mauritius. Therefore, this study was proposed with the following objectives:

- To assess fruit and vegetable intake, energy intake, physical activity level, body mass index (BMI), nutrition knowledge and attitudes of adults before and after a nutrition education.
- To determine factors associated with nutrition knowledge.
- To evaluate the efficacy of the nutrition education strategy.

## 2. Materials and Methods

**Figure 1:**
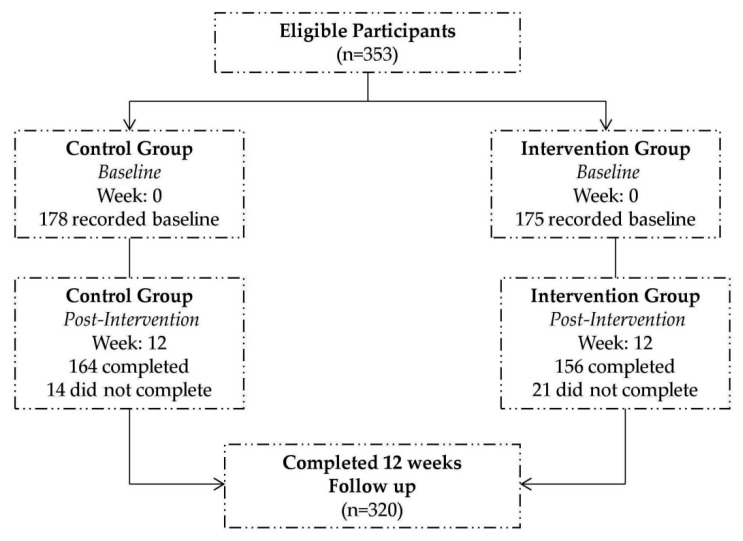
Consort diagram for recruitment of participants.

The study used a pre- and post-test design consisting of pre- and post-survey with random assignment of two community centers to the intervention group (IG), receiving NE lessons, individual telephone counseling and educational tools and another two community centers to the control group (CG) which did not receive any lessons. Both groups completed pre- and post-test after 12 weeks. Community centers were contacted officially and were randomly selected among those granting permission to conduct the survey. Name of the centers were kept anonymous as requested by the authorities. Upon obtaining informed consent from participants, a total of 353 Mauritian adults aged between 19 and 55 years; generally healthy and able to understand Creole language were recruited. Among them, 178 participants were in the (CG) and 175 participants were in the (IG). Participants were excluded if they were pregnant, were older than 55 years or were suffering from mental disability. After 12 weeks, due to drop-outs (unexplained reasons); a total of 320 participants completed the entire intervention study (Figure 1).

At baseline, participants completed a self-reported questionnaire which consisted of sociodemographic variables and a food frequency questionnaire (FFQ) adapted from the Nutritional Epidemiology group at Leeds University [26]. The questionnaire included 67 items classified under the headings: carbohydrates (grains and tubers), beans/pulses, meat and eggs, seafood, soybeans and products, dairy products, fruits, vegetables, processed foods, snacks high in fat and sugar and beverages. For each FFQ item, participants could select from 7 frequency categories and scores were allocated as shown in the Table 1 [27].

Information pertaining to sociodemographic data was related to age, gender, marital status, employment status, educational level and monthly household incomes. Age groups of adults were classified into the following categories (i) 19–25 years (ii) 25–45 years (iii) 45–55 years. The household income per month was classified as follows (i) low income (less than $138 to $276) (ii) middle income ($277 to $830) and (iii) high income (above $830). Questions on nutrition knowledge (NK) were adapted from the general nutrition knowledge questionnaire for adults developed by Parmenter and Wardle [28] which has been previously used to evaluate knowledge in a large community sample. A score of 1 was allocated for a good answer and 0 for a wrong answer or “not sure” on a total score of 15. The total score was divided into tertiles, with the lowest one receiving an “insufficient NK”, the medium one a “quite good NK” and the highest one was given a “good NK”. Education level was stratified as follows: Low- None, Primary Level; Medium- Secondary Level; High-Tertiary Level [29].

The Attitudinal part comprised of statements adapted from Social Science Research Unit 2009 [30]. It consisted of three main sections with the following statements:

(1) Section 1: “I intend to try healthier eating”
(2) Section 2: “Healthier eating” means
(3) Section 3:”How far do you agree with the following statements…?”

Data was interpreted in a 5-point Likert scale which ranged from (a) strongly disagree; (b) disagree; (c) neutral; (d) agree and (e) strongly agree. To present and compare the Likert data efficiently individual responses were assigned a score of 1 to 5 and mean score for each item was calculated. During scoring, the direction of the question was considered whereby responses of “Strongly Disagree or disagree” for negative statements and “Strongly Agree and Agree” for positive statements both received higher score.

Physical activity level was assessed using questions modified from the Global Physical Activity Questionnaire developed by the World Health Organisation [31]. The Metabolic Equivalent of Task (MET) score was calculated and was categorized into low, moderate and high physical activity level. Besides, throughout both the pre and post survey, a 15-min interview was conducted with each participant to gather information for a 24 h diet recall adapted and designed from EPIC-Norfolk nutritional methods [32]. Participants were phoned and were followed for 3 days over 3 weeks and mean energy intake for the 3 days was calculated to reduce errors. Due to lack of an established Food Composition Table in Mauritius, that of Tanzania Food Composition Table [33] was used to calculate the energy intake. Anthropometric measurements were taken by the investigator. Body Mass Index was calculated according to WHO classification [34]. After 12 weeks, the same questionnaire was used to re-evaluate the variables assessed for both the CG and IG. Other questions were included in the post-intervention questionnaire to find out whether the participants found the nutrition education helpful and to identify potential barriers and motivators in implementing the strategy. Low educational level adults were assisted by the investigator who translated the questions in “Creole” language and personally noted down their answers.

### 2.1. Nutrition Education Strategy

**Figure 2:**
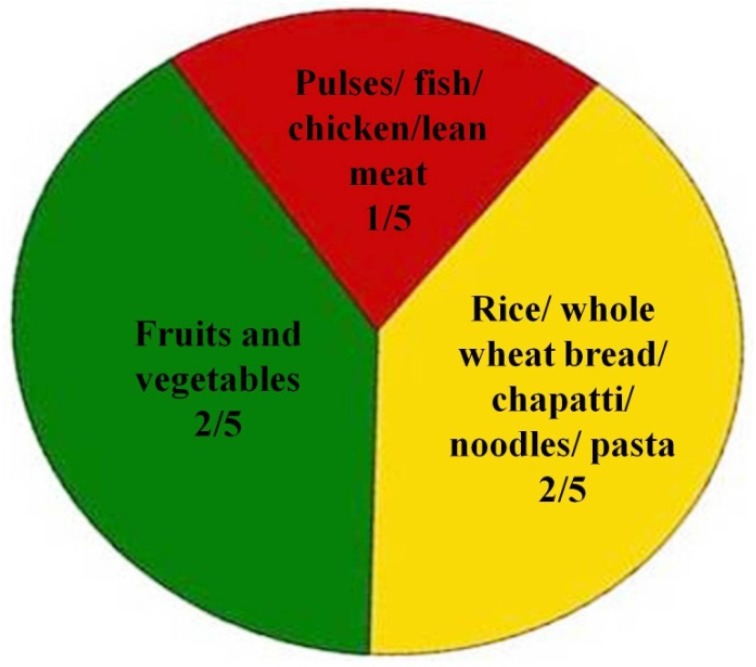
Mauritian Plate Model. Source: Ministry of Health and Quality of Life, Mauritius.

The nutrition education strategy was designed partially from the adult learning theory. It consisted of four steps: (a) assessing the needs of the learner (b) setting educational objectives (c) choosing and using a variety of methods (d) assessing that learning occurred [17]. Learning styles of adults were identified from a pilot study (visual, auditory, collage, *etc.*), objectives that focus on what learner will do with the contents of the nutrition education strategy in order to learn it were set and the intervention was designed accordingly. Participants in the IG attended a multicomponent nutrition education entitled “Healthy eating For a Healthy Living” using 3 modes (lecture/talk, leaflet, picture collage). The adults were gathered in 2 sessions for 2 h duration on average. 2 h duration of a session was considered as sensible as previously done by Liu *et al.* [35]. Creole language as the national language was used. Both sessions were held in groups and were carried out by the investigator. Comprehension of the educational tools was pretested in a sample of 15 adults before the intervention. The first session comprised of a lecture guided by a poster and covered topics that addressed overall dietary quality, including: (a) identifying a preferable overall distribution of types of food in a diet using the plate model from the Ministry of Health and Quality of Life (Figure 2); (b) increasing consumption of fruits and vegetables and whole grain products; (c) promoting physical activity; (d) discouraging consumption of foods high in fat and sugar, (e) providing alternatives to low nutrient snacks. Question and answer sessions were conducted along the lecture to enable active participation. The second session consisted of a brief summary of all topics discussed during the previous session and a picture collage was introduced where participants had to use pictures and place them in appropriate portions on a designed plate model. At the end of the second session, a leaflet consisting of topics discussed above together with a “sample menu” of breakfast, lunch, dinner and “alternatives to junky snacks (low nutrients snacks)” featuring mainly Mauritian snacks was distributed to the participants as take home lessons to enhance their understanding of the whole sessions.

### 2.2. Ethical Approval

Ethical approval was granted by appropriate research committees.

### 2.3. Statistical Analysis

Means and standard deviations were calculated for all the variables analyzed. The statistical analyses were carried out using the Statistical Package for the Social Science (SPSS)^®^ version 20.0 (IBM, Armonk, New York, NY, USA). The IG and CG were compared descriptively with respect to sociodemographic characteristics. The categorical variables were expressed as percentages. While controlling for confounding factors (age, sex, BMI, baseline measures), ANCOVA was utilized to examine changes in dietary intakes from baseline to 12 weeks after intervention between IG and CG. Secondary analyses were conducted for IG only. Pre-post differences were assessed using Paired Sample *t*-tests. Independent Samples *t*-test/One way Anova was used to assess consumption of fruits and vegetables at baseline. One way Anova was used to assess factors associated with nutritional knowledge. Significance was set a *priori* at *p* < 0.05.

## 3. Results

### 3.1. Participants’ Profiles

Table 2 depicts the profiles of the participants.

### 3.2. Intervention and Control Group at Baseline

In general, there was a high consumption of carbohydrates foods and vegetables among adults in both groups. As shown in Table 3, a high fruit and vegetable intake was observed for females. In contrast, income level, educational level, nutritional knowledge, physical activity level, BMI and access to food commodities did not have an effect on fruit and vegetable intake.

The mean energy intake of the participants was 1997 Kcal and 1918 Kcal for the CG and IG, respectively. The caloric intake did not differ statistically between the two groups. According to gender, male reported higher mean energy intake than females in both CG (M = 2081 Kcal for male; 1967 Kcal for female) and IG (M = 2023 Kcal for male; M = 1876 Kcal for females). Results revealed a mean BMI of 24.4 Kg/m^2^ and 23.1 Kg/m^2^ for the CG and IG respectively and the differences were found to be significant (*p* = 0.009). Overall most adults reported low physical activity level (51.1% CG; 50.9% IG) and are not meeting the physical activity guidelines for adults. 32.0% in CG and 29.1% in IG had moderate physical activity level while only 16.9% and 20.0% in CG and IG respectively reported high physical activity level. The mean nutritional knowledge score was 9.47 ± 5.22 and 9.74 ± 2.00 in CG and IG, respectively. The two groups did not differ in terms of nutrition knowledge (*p* = 0.538). The results demonstrate that majority of participants (66.9% CG; 62.3% IG) had quite good nutrition knowledge. 26.4% in CG and 37.1% in IG demonstrated good nutrition knowledge while 6.7% in CG and 0.6% in IG reported insufficient nutrition knowledge. Results revealed that nutrition knowledge was significantly related to age (*p* = 0.000), gender (*p* = 0.039) and education level (*p* = 0.000). In contrast income level had no significant influence on nutrition knowledge (Table 4).

### 3.3. Post Intervention Changes

Mean dietary frequency scores at baseline and after 12 weeks of intervention among IG and CG are presented in Table 5. After controlling for potential confounders such as age, sex, BMI and baseline measures, a significant increase in fruit intake and a decrease in snacks high in sugar and fat could be observed among IG (*p* < 0.05) compared to CG. Further statistical analyses revealed an increase in fruit score from 2.95 ± 1.84 to 3.79 ± 1.85 while snacks high in sugar and fat score decreased from 2.20 ± 2.06 to 1.56 ± 1.69 in the IG. No intervention effect was observed for vegetables (*p* = 0.659) and other food groups. No significant changes in mean energy intake (*p* = 0.507 in CG; *p* = 0.929 in IG) and BMI (*p* = 0.760 in CG; *p* = 0.768 in IG) was experiential. Physical activity level was still low among adults in both study groups. The percentage of participants with high physical activity level decreased from 20.0% to 12.7% in IG; while, those with moderate physical activity level showed an increase from 29.1% to 34.4% in the CG. Moreover, a significant increase in nutrition knowledge from pre-test (8.89 ± 1.97) to post-test (10.19 ± 2.13) and in scores for nearly each item of the attitude statements (*p* < 0.05) for the IG was also observed.

### 3.4. Opinions, Barriers and Motivators of the Nutrition Education Strategy

Table 6 shows perceived opinions on the nutrition education strategy. Participants identified major barriers in implementing the nutrition education strategy to be “taste preference” (49.4%) followed by “lack of motivation” (37.2%), “traditional food preparation at home” (36.5%) and “no time” (32.7%). Availability (6.4%) and affordability (10.9%) were the least reported barriers in this study. Common motivators to implement the nutrition education strategy reported in the investigation were “to protect myself from diseases” (75.6%) followed by “to maintain my weight” (67.9%) and “for my own personal look” (55.8%).

## 4. Discussion

The present study examined the impact of the nutrition education intervention among Mauritian adults using the Adult Learning Theory [17]. A significant increase in fruits, nutritional knowledge, attitudes towards healthier eating and a significant decrease in snacks high in sugar and fat was observed (*p* < 0.05). No intervention effect was found for vegetables intake, energy intake and BMI (*p* > 0.05). Fruit and vegetable intake was significantly associated with gender (*p* < 0.05). Nutrition knowledge was significantly correlated with age, gender and education level (*p* < 0.05). Taste was the main barrier to the application of the nutrition education strategy.

### 4.1. Changes in Dietary Intake

Vegetable intake among all participants at baseline were relatively high followed by carbohydrates, fruits, dairy products, snacks high in sugar, sugar sweetened beverages, meat and eggs, processed foods, pulses, seafood and soybeans (Table 5). However, whether adults are meeting current recommendations are not known. High vegetable intake score can be attributed to a decrease in price of fresh vegetables (−28%) [36] and an increased globalization. Other plausible reasons for high vegetable intake in Mauritius are easy access to variety of vegetables [37], affordability and availability of considerable amount of canned and frozen vegetables [38]. A statistically significant relationship was observed between fruit and vegetable intake and gender (*p* = 0.022). Male had lower intake than female (Table 3). This finding is as reported by Emanuel *et al.* [39] where gender was strongly associated with total fruit and vegetable intake. A possible reason may be that fruits and vegetables are more associated with femininity and are eaten with more delicacy and patience while male tends to go for foods such as meat or hearty portion sizes foods [40]. Studies have reported that females feel more energetic when they consume healthier foods like fruits and vegetables [41] and are more interested in healthy eating compared to men [42]. Worldwide, across 24 countries 57% of men and 69% of women reported to eat fruit daily and gender differences in vegetables intake exceeded by 15% in countries like Norway, Denmark, Finland and Germany [43]. However some studies have shown that men tend to underestimate their fruit and vegetable consumption while the opposite applies to women [44]. At post intervention, a statistically significant increase in fruit intake (*p* = 0.000) and decrease in snacks high in sugar and fat (*p* = 0.003) in the IG was observed. No intervention effect was found for vegetable intake (*p* = 0.659). This finding is in agreement with previous research that showed positive changes in fruits intake but no effect on vegetable intake after implementation of nutrition education [45]. Different types of food have different adjustment periods [23] hence changes in fruit intake can be achieved more quickly compared to changes in vegetable intake. Moreover, most vegetables require preparation skills compared to fruit which is eaten raw. Recently Chapman *et al.* [24] demonstrated that a combined instruction was successful in increasing fruit intake but not vegetable intake which is equivalent to the present nutrition education intervention. The result of the study is inconsistent with other research showing an increase in both fruits and vegetables [46]. This inconsistency may be because vegetable intake was already high at pre intervention. Besides, most studies aiming to increase fruit and vegetable considered the latter as one entity and concluded positive intervention effects [47]. This research also found a significant decrease in snacks high in sugar and fat (*p* = 0.003). Similar findings have been reported in interventions done in children aiming weight loss [48] or studies targeting cardiovascular risk factors by reducing energy dense foods [49] since snacks high in sugar and fat contribute to substantial calories [50]. Similarly it can be suggested that this intervention effect was observed among adults because of their high level of concern with rising rates of diabetes and obesity in Mauritius.

### 4.2. Mean Energy Intake and BMI

The mean energy intake of Mauritian adults is 1958 Kcal. Men and women had a mean energy intake of 2051 Kcal and 1923 Kcal respectively. The results are found to be close to that of the Asian Indians living in UK reporting 2134 Kcal among men and 2067 Kcal among women [51]. No significant changes in mean energy intake were observed after intervention while other studies demonstrated positive changes in energy intake [20]. This inconsistency may be attributed to varying methodologies, timing of data collection and intervention strategies used (for instance, whether targeting low fat diet or healthy eating) and target population (e.g., whether targeting slim, overweight or obese participants). In this study, focus was not at reducing energy intake rather promoting healthy dietary habits based on fruit and vegetable intake and reduction of energy dense foods. It would be unwise to recommend reducing energy intake as it falls within or slightly below the recommended intakes. Mean BMI in this study was 23.8 ± 4.64 Kg/m^2^. This finding is consistent with previous research reporting a normal BMI of 23.29 ± 4.58 Kg/m^2^ [52] and 23.1 Kg/m^2^ [53] in adults from Lativa and UK, respectively. In contrast a higher BMI was registered among Australian [54] and Lithuanian adults [55]. This finding is imperative for policy and intervention strategies that aim to shift a risk factor in a favorable path by applying effective intervention to an entire population [56]. However it is also true that screening of BMI alone is not ideal to predict obesity prevalence since Chinese people with BMI < 25 Kg/m^2^ were at high risk for obesity [57]. In contrast to other research demonstrating positive changes in BMI, no significant change in BMI was observed at post intervention. This is not surprising as participants had normal BMI at baseline. Most studies involving nutrition intervention have focused on overweight or obese participants [58].

### 4.3. Mean Nutritional Knowledge (NK)

The foregoing results reveal that majority of participants (64.6%) had quite good nutrition knowledge before the nutrition education intervention. This finding is essential as nutrition knowledge is a major determinant of dietary intake among adults [59]. The investigation resulted in a statistically significant relationship between nutrition knowledge and age (*p* = 0.000), gender (0.039) and education level (*p* = 0.000). Previous studies also demonstrated higher nutrition knowledge among younger adults [60], males [61] and those with higher education level [62]. Compared to the CG, a statistically significant increase in nutrition knowledge (*p* = 0.000) was observed in the IG after the nutrition education intervention. Other studies also reported significant increase in nutrition knowledge among adults [63]. Results reveal that the intervention notably impacted on nutrition knowledge which is an integral component of health literacy and low health literacy is associated with poor health outcomes [64]. In the present investigation, no significant relationship was observed between fruit and vegetable intake and nutrition knowledge after the nutrition education intervention (*p* < 0.05). Previous studies also demonstrated a weak correlation between increased nutrition knowledge and dietary changes [65]. These findings are encouraging as they demonstrated a significant impact on nutrition knowledge after an intervention. Planning of effective nutrition education strategies necessitate better formulation of the ways through which nutrition knowledge influences dietary change. 

### 4.4. Attitudes towards Healthier Eating

Results revealed a statistically significant increase in attitudes towards healthier eating scores (*p* < 0.05) after the nutrition education intervention. The results are consistent with other research reporting positive attitudes after an intervention [66]. These findings have important implications as they can help to identify appropriate target groups in relation to their differing attitudes to plan effective nutrition education strategies. At post-intervention a significant proportion of adults in both study groups agreed that they “intend to try healthier eating”. Such result suggests that majority perceived themselves to try healthy eating but whether these perceived changes in attitudes have translated into action is not known. Recent studies have suggested that changing diet is challenging and the ability to turn intentions into actions can be related to individual differences [67]. Adults in this study do not perceive the need to limit use of oil and solid fat like butter and maintain weight, believing they are already doing so, and this action can be called “optimistic bias” [68]. This occurrence of “optimistic bias” has useful implications, as lack of awareness of personal behavior is related with a low motivation to change [69].

### 4.5. Barriers and Motivators to Nutrition Education Strategy

Major barrier to the application of the nutrition education strategy as cited by the participants is “taste preference”. Similar barriers have been reported in previous research [70]. Major factor perceived as motivators is “to protect myself from diseases”. Other studies have identified personal appearance, fit in clothes, social support and friends [71] as potential motivators to healthy eating. As indicated by the “health belief model”, perceived barriers reflect a person’s opinion about the tangible cost of a behavior [72]. Current results indicate that unhealthy choices and low fruit and vegetable intake could be based on adults’ personal taste preferences which is an important predictor of food choice and is slow to change [73].

## 5. Limitations of the Study

Major limitations of the current study are as follows: (i) the nutrition education intervention was conducted only over a period of 12 weeks and hence no significant changes in anthropometric measurements for the intervention group could be found; (ii) Cultural traditions may have a profound influence on the food choices of each ethnic group in Mauritius and these have not been considered; (iii) Mean energy intake of 3 days may not be representative of long term dietary habits which are sometimes subjected to festival changes.

## 6. Conclusions

Participants who attended the nutrition education intervention substantially reported changes in dietary behaviors, knowledge and attitudes. Mean energy intake and body mass index were in the recommended range for adults but low physical activity level was demonstrated among Mauritian adults. After intervention, a significant increase in fruits, nutrition knowledge, attitudes towards healthier eating and a significant decrease in snacks high in sugar and fat was observed. No intervention effect in vegetables intake, energy intake, body mass index and physical activity level was recorded. Factors influencing nutrition knowledge were age, gender, and education level. Taste was the main barrier to the application of the nutrition education strategy. The current study is the first nutrition education intervention on fruit and vegetable intake and energy intake among Mauritian adults. However, findings add to existing research that a nutrition education program is a promising strategy to improve dietary behaviors. Results are helpful to health practitioners and health educators when counseling or designing nutrition interventions for adults. In addition, this study provides baseline evidence to show that healthy lifestyle education programs for adults should be tailored to address low physical activity level in this target population. Findings also indicate that nutrition education intervention should target specific subgroups of populations such as older adults in the age group of 45–55, those with low education level as well as males who have a low intake of fruits and vegetables. Additional studies should look at the effect of nutrition education intervention and exploit various theories in a more diverse and larger sample of Mauritian adults so that health educators can deem the use of theory-driven approaches in their intervention programs.

## Figures and Tables

**Table 1:** Food Frequency Scores.

Frequency	Score
Never or less than once per month	0
Monthly	0.033
Twice per month	0.08
Once per week	0.14
2–3 times per week	0.5
Once daily	1
2–3 times daily	2

**Table 2:** Percentage distribution of sample profiles.

Characteristics	Categories	* CG (*n* = 178) %	** IG (*n* = 175) %
Age (years)	19–25	16.1	16.0
	25–45	66.1	66.3
	45–55	17.8	17.0
Gender	Male	25.8	28.6
	Female	74.2	71.4
Marital Status	Single	20.2	28.6
	Married	73.0	68.5
	Separated	1.1	0
	Divorced	2.2	0.6
	Widowed	3.4	2.3
Employment Status	Full time employed	12.9	19.0
	Part time employed	3.4	4.0
	Self employed	2.2	2.9
	Unemployed	78.8	72.0
	Retired	1.7	2.0
Education Level	Low	7.5	7.4
	Medium	46.6	45.7
	High	48.9	46.9
Total Household income	Low	28.1	26.3
	Moderate	57.3	56.2
	high	14.6	17.5

* CG: Control Group; ** IG: Intervention Group; Data is expressed as *n* (%).

**Table 3:** Consumption of fruits and vegetables by demographic variables at baseline.

Independent Variables		Mean ± SD	Std. Error of Mean	*p* Value
Gender	Male	6.32 ± 3.43	0.35	0.022 *^,a^
	Female	7.27 ± 3.49	0.22	
Age (years)	19–25	6.92 ± 3.76	0.32	0.744
	25–45	6.94 ± 3.38	0.30	
	45–55	7.25 ± 3.27	0.34	
Income Level	Low	6.69 ± 3.61	0.36	0.534
	Moderate	7.32 ± 3.45	0.40	
	High	7.10 ± 3.45	0.26	
Education Level	Low	7.38 ± 4.11	0.90	0.272
	high	6.73 ± 3.50	0.25	
	Medium	7.33 ± 3.39	0.28	
Nutrition Knowledge	Insufficient	6.42 ± 3.73	1.03	0.792
	Good	6.96 ± 3.62	0.34	
	Quite Good	7.07 ± 3.43	0.23	
Physical Activity Level	Low	6.59 ± 3.39	0.25	0.051
	Moderate	7.61 ± 3.73	0.36	
	High	7.18 ± 3.27	0.41	
Body Mass Index (BMI)	Underweight	6.41 ± 3.21	0.50	0.424
	Overweight	6.72 ± 3.48	0.39	
	Normal	7.17 ± 3.39	0.24	
	Obese	7.45 ± 4.25	0.68	
Access to Food Commodities	Easy	7.02 ± 3.59	0.42	0.761
	Difficult	6.83 ± 2.29	0.20	

* Statistically significant relationship (*p* < 0.05); ^a^ independent samples *t* test.

**Table 4:** Factors associated with nutrition knowledge.

Parameters	Categories	Mean ± SD	Std. Error of Mean	*p* Value
Age (years)	19–25	2.42 ± 0.50	0.04	0.000 *^,a^
	25–45	2.29 ± 0.54	0.05	
	45–55	2.06 ± 0.49	0.05	
Gender	Male	2.38 ± 0.55	0.06	0.039 *^,b^
	Female	2.25 ± 0.51	0.03	
Income Level	Low	2.26 ± 0.54	0.05	0.311
	Moderate	2.32 ± 0.50	0.04	
	high	2.21 ± 0.56	0.06	
Education Level	Low	2.05 ± 0.38	0.08	**0.000** *^,a^
	Medium	2.15 ± 0.52	0.04	
	high	2.40 ± 0.51	0.04	

* Statistically significant relationship (*p* < 0.05); ^a^ ANOVA; ^b^ independent samples *t* test; SD: Standard deviation.

**Table 5:** Mean frequency scores of food group intakes in intervention (*n* = 175) and control (*n* = 178) group and ANCOVA analysis after controlling for potential confounders.

Food Group Intakes	Mean ± SE		Adj. Mean ^a^ 95% CI	Adj. Mean Dif ^b^ (95% CI)	F-stat (d*f*)	*p* Value ^a^
	Baseline	After 12 Weeks				
**Carbohydrates (Grains and tubers)**				0.17 (−0.79, 0.45)	1.58 (11, 309)	0.600
Intervention	3.84 ± 0.11	3.93 ± 1.87	3.74 (3.12, 4.14)			
Control	3.60 ± 0.11	3.61 ± 1.49	3.62 (3.09, 3.82)			
**Beans/Pulses**				0.001 (0.16, 0.17)	1.39 (11, 309)	0.990
Intervention	0.64 ± 0.39	0.67 ± 0.47	0.63 (0.7, 0.72)			
Control	0.64 ± 0.39	0.60 ± 0.46	0.64 (0.48, 0.71)			
**Meat and eggs**				0.14 (−0.40, 0.11)	0.92 (11, 309)	0.272
Intervention	0.95 ± 0.06	0.84 ± 0.86	0.98 (0.55, 1.03)			
Control	0.61 ± 0.56	0.59 ± 0.50	0.60 (0.53, 0.78)			
**Seafood**				0.15 (−0.34, 0.05)	1.53 (11, 309)	0.133
Intervention	0.52 ± 0.04	0.53 ± 0.63	0.52 (0.34, 0.68)			
Control	0.36 ± 0.40	0.35 ± 0.37	0.36 (0.26, 0.44)			
**Soybeans and products**				0.06 (−0.17, 0.29)	2.59 (11, 309)	0.590
Intervention	0.22 ± 0.47	0.23 ± 0.50	0.22 (0.05, 0.33)			
Control	0.26 ± 0.05	0.26 ± 0.74	0.26 (0.07, 0.43)			
**Dairy Products**				0.10 (−0.67, 0.47)	2.56 (11, 309)	0.726
Intervention	2.31 ± 1.11	2.12 ± 1.80	2.29 (1.46, 2.45)			
Control	2.19 + 1.11	2.08 ± 1.31	2.16 (1.55, 2.18)			
**Fruits**				0.58 (−1.20, 0.04)	1.23 (11, 309)	<0.05
Intervention	2.96 ± 0.13	3.79 ± 1.85	2.95 (3.01, 4.01)			
Control	2.70 ± 0.12	2.94 ± 1.53	2.68 (2.61, 3.36)			
**Vegetables**				0.09 (−0.97, 0.78)	1.74 (11, 309)	0.837
Intervention	4.18 ± 0.17	4.23 ± 2.40	4.11 (3.56, 4.88)			
Control	4.19 ± 0.17	4.23 ± 2.40	4.11 (3.49, 4.65)			
**Processed Foods**				0.27 (−0.58, 0.04)	1.49 (11, 309)	0.08
Intervention	0.83 ± 0.08	0.71 ± 0.95	0.82 (0.42, 0.95)			
Control	0.48 ± 0.07	0.47 ± 0.72	0.46 (0.24, 0.60)			
**Snacks High in Sugar and Fat**				0.19 (−0.36, 0.74)	1.15 (11, 309)	<0.05
Intervention	2.12 ± 0.13	1.64 ± 1.28	2.20 (0.99, 1.93)			
Control	1.70 ± 0.13	1.56 ± 1.69	1.66 (1.31, 1.94)			
**Beverages**				0.01 (−0.32, 0.30)	2.71 (11, 309)	0.953
Intervention	1.36 ± 0.07	1.34 ± 0.90	1.35 (0.99, 1.48)			
Control	1.30 ± 0.07	1.33 ± 0.80	1.28 (1.02, 1.41)			

^a^ adjusted mean using ANCOVA after controlling for age, sex, BMI and baseline measures for each variable; ^b^ Bonferroni adjustment for 95% CI for difference; SE: Standard error of the mean; Adj: adjusted; Dif: difference.

**Table 6:** Opinions on the Nutrition Education Strategy.

Questions	Post Intervention (*n* = 159) (%)		
Liked session?	71.2% very much	28.8% slightly	0% not
Opinions? It was:	0% Annoying	0% Difficult	51.3%Encouraging
	0% Boring	34.6% Simple	71.2% Interesting
	0% Confusing	60.3% Healthy	24.4% new
	5.1% Time consuming	42.9% Enjoyable	
Heard new information?	29.5% A lot	64.1% A few things	6.4% none
Important to have received the information?	66.7% very	32.1% slightly	1.3% Not
Intend to change?	80.8% Yes	19.2% No	
